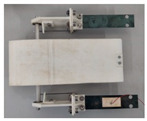# Experimental Investigation on a Novel Airfoil-Based Piezoelectric Energy Harvester for Aeroelastic Vibration

**DOI:** 10.3390/mi11080725

**Published:** 2020-07-26

**Authors:** Xiaobiao Shan, Haigang Tian, Han Cao, Ju Feng, Tao Xie

**Affiliations:** State Key Laboratory of Robotics and System, Harbin Institute of Technology, Harbin 150001, China; 18B908022@stu.hit.edu.cn (H.T.); ershugm@gmail.com (H.C.); fengju223@163.com (J.F.)

**Keywords:** nonlinear aeroelastic vibration, limit-cycle oscillation, piezoelectric energy harvester, harvesting performance, field testing

## Abstract

This paper presents a novel airfoil-based piezoelectric energy harvester (EH) with two small square prisms attached to an airfoil. This harvester can achieve a two degree-of-freedom (DOF) plunge–pitch motions. Several prototypes of energy harvester were fabricated to explore the nonlinear aerodynamic response and the output performance in a wind tunnel. The experimental results showed that the longer the flexible spring was, the lower the critical velocity and frequency of the harvester were, and the better aerodynamic response and output performance could be achieved. The initial disturbance, the following limit-cycle oscillation, and the ultimate chaos of nonlinear response occurred, as increasing airflow velocity was increased. The overall output performance of the harvesters with a flexible spring having a thickness of 1 mm outperformed than that of the harvesters with a flexible spring having a thickness of 0.5 mm at a higher airflow velocity, while the tendency was opposite at a lower velocity. An optimum output voltage of 17.48 V and a power of 0.764 mW were harvested for EH-160-1 at 16.32 m/s, which demonstrated it possessed better performance than the other harvesters. When the capacitor was charged for 45 s and directly drove a sensor, it could maintain working for 17 s to display temperature and humidity in real time.

## 1. Introduction

Vibration-based energy harvesters which are usually located at remote or inconveniently accessible places, are considered as promising independent power sources for low-power electric devices and have been studied [1,2,3]. Different from the most commonly utilized chemical batteries, energy harvesters function like power generators, endlessly harvesting energy from the ambient environment. Possible vibration-to-electric conversion mechanisms include piezoelectric transduction [4,5,6,7,8], electromagnetic transduction [9,10], electrostatic transduction [11,12], and triboelectric transduction [13,14]. Because of the advantages of easy production, high power density, and no electromagnetic interference [15,16,17], piezoelectric transduction has become a research topic globally over the last decades.

Flow-induced vibration exists everywhere in nature and reserves tremendous energy, including vortex-induced vibration [18,19,20], wake-induced vibration [21,22], flutter-induced vibration [23,24,25], and galloping-induced vibration [26,27,28]. Energy harvesting from flutter-induced vibration has been extensively investigated in recent decades [29,30,31,32]. For example, Erturk and his coauthors [33,34] designed a piezoelectric-inductive harvester and investigated in detail the relation between the flutter velocity and the maximum power. Kwon [35] proposed a T-shape bimorph cantilever beam for energy harvesting from a flutter and hastened the occurrence of the flutter at a low airflow velocity. It was found that a continuous peak power of 4 mW was obtained at 4 m/s. Inman and his coauthors [36,37,38] designed a piezoelectric composite wing for energy harvesting and gust alleviation simultaneously in a small unmanned aerial vehicle, which achieved the better performance of energy harvesting and gust alleviation during normal flight and gust response. Aquino et al. [39] proposed the integration of a wind-induced flutter energy harvester (WIFEH) into the built environment, and experimental investigation and simulation analyses were performed to explore the effects of airflow velocities and incoming airflow angles on open-circuit voltages, short-circuit currents, and output powers. Dunnmon et al. [40] exploited the nonlinear limit-cycle oscillation of an aeroelastic energy harvester with a cantilever beam attached to the trailing edge of a fixed airfoil, and the characteristic of the limit-cycle oscillation and the harvesting performance were investigated both experimentally and numerically. Zhao et al. [41] designed a novel flutter energy harvester by attaching a beam stiffener to a substrate, which achieved a better harvesting performance over conventional designs without a beam stiffener.

In addition, Abdelkefi et al. [42,43] explored the performance enhancement of wing-based piezoaeroelastic energy harvesting by freeplay nonlinearity, and the freeplay gap can reduce the flutter onset speed through a subcritical instability and capture energy at a low airflow velocity. Li et al. [44] and Naseer et al. [45] adopted the nonlinearity of a magnetic force to enhance the energy harvesting performance, reduce the cut-in airflow velocity and broaden the operating frequency, which demonstrated that the designed energy harvesters manifested better performance. Zhu et al. [46] proposed an electromagnetic transducer by attaching a wing to a cantilever spring, which demonstrated better performance at a lower airflow velocity. Orrego et al. [47] designed a novel flutter energy harvester with an inverted flag and studied the influence of geometrical parameters of the flag on the flapping behavior and the harvesting performance. The sustained power of about 0.4 mW/cm^3^ was harvested at 3.5 m/s. Zhou et al. [48] proposed a novel Y-shaped bi-stable energy harvester with two curved wings and a tip magnet, which demonstrated that the harvester could execute snap-through and reach coherence resonance in a wide range of airflow velocity. Dowell and his coauthors [49,50,51] investigated a flutter and the limit-cycle oscillation of a fixed cantilever beam. An aerodynamic model was developed, and the effects of airflow velocity on aeroelastic vibration and limit-cycle oscillation were obtained. Xiang and his coauthors [52,53] proposed a novel pitch–plunge–leadlag airfoil-based piezoaeroelastic energy harvester and investigated numerically the effects of the structural parameters of the harvester on the aeroelastic characteristics and the output power. The results showed that the aeroelastic response and the output power of the harvester in the plunge motion were better than those in the leadlag motion. Marqui and his coauthors [54,55] investigated a plunge–pitch aeroelastic harvester by a simulation method and explored the effect of system parameters on the flutter speed and the harvesting performance.

As can be seen from the above reports, many flutter-induced energy harvesters with a single and specific flexible spring were proposed and designed to explore the aeroelastic response and the harvesting performance. However, to the best of our knowledge, flutter-induced energy harvesters with various structural parameters of a flexible spring have rarely been reported in the literature to date. The flexible spring and the small square prism attached to the airfoil could improve the aerodynamic force, which play a vital role in harvesting characteristics. It is necessary to further explore the effects of the structural parameters of the flexible spring, airflow velocities, and external load resistances on aerodynamic response and output performance, with the aim of enhancing the comprehensive characteristics of the proposed harvester. The harvester utilizes an airfoil as an interference body for exploring the output characteristic compared with circular, square, and triangle ones. In addition, the harvester could stimulate two degree-of-freedom (DOF) plunge–pitch motions, which may be considered for applications in future micro or unmanned airfoil aircrafts.

Therefore, this paper presents a novel piezoelectric energy harvester with a small square prism attached to an airfoil and aims to investigate experimentally the nonlinear aeroelastic vibration response and the energy harvesting performance of the proposed harvester in an airflow. Because of the existing difference between experimental and theoretical results, the theoretical model was not included in this paper. The modeling of the harvester is introduced in Section 2. A wind tunnel experimental system is designed and fabricated. The experimental analyses are performed to explore the aeroelastic response and the harvesting performance in Section 3. The conclusions of this work and future outlook are drawn in Section 4.

## 2. Modeling and Fabrication of the Harvester System

### 2.1. Modeling of the Harvester

**Figure 1:**
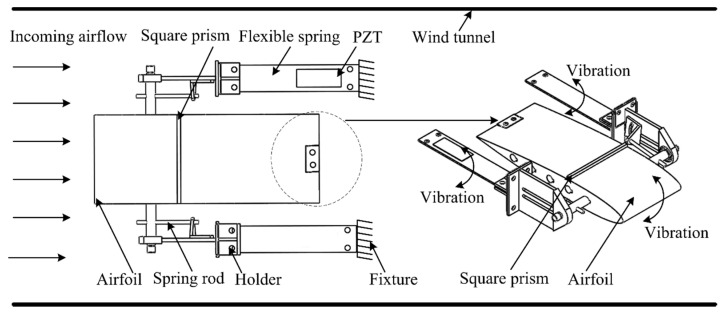
Schematic of a piezoelectric energy harvester.

Figure 1 illustrates the schematic of a piezoelectric energy harvester with a small square prism attached to an airfoil in a uniform airflow. This harvester system mainly simulated two DOF plunge–pitch motions for capturing the aeroelastic vibration of the airfoil. This harvester included an airfoil, two flexible springs, two spring rods, and two square prisms, so the flexible spring harvester was composed of a piezoelectric patch and a flexible spring. Due to the restriction of the flexible spring in the plunge and the flexible spring rod in the pitch, two DOF plunge–pitch motions were shown. When subjected to an incoming aerodynamic airflow, the airfoil underwent aeroelastic vibration in a transverse direction. The negative aerodynamic damping increased with the airflow velocity, gradually exceeded the structural damping and eventually resulted in the overall negative damping of the harvester. Therefore, it turned into a flutter with a constant amplitude and self-excitation. The small square prism attached to the airfoil at a three-eighths chord aimed to change the flow field, increased the windward area, amplified the aerodynamic force, promoted the aeroelastic instability of the harvester and thus enhanced the harvesting ability. Therefore, the flexible spring and the small square prism attached to the airfoil actually exerted an important effect on the aerodynamic response of the harvester. This manuscript focused on the effect of the flexible spring on the aeroelastic response and the harvesting performance, and the effect of the square prism was not considered.

### 2.2. Experimental Setup

**Figure 2:**
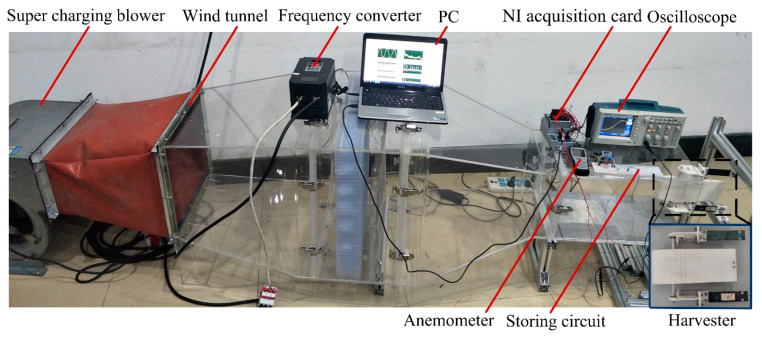
Wind-tunnel experimental system.

Figure 2 shows a wind tunnel experimental setup with a cross-sectional dimension of 300 × 300 mm^2^ at the testing section. An airflow velocity of 22.73 m/s was obtained with a frequency converter (V88, Shenzhen Wanchuanda Technology Co., Ltd., Shenzhen, China) working at 50 Hz. The airflow velocity after flowing around the contraction section was nearly uniform and stable. The system was mainly composed of a wind channel, a supercharging blower (LKW315L-4, Shandong Qineng Ventilator Co., Ltd., Zibo, China), an airfoil-based harvester, an anemometer (AR866A, Smart Sensor, Hong Kong, China), an energy-collecting and -storing circuit, and a data-acquiring and -processing system. The energy harvester was placed symmetrically at the testing section by a base fixture. The data-acquiring and -processing system consisted of an NI acquisition card (NI 9229, National Instruments, Austin, TX, USA), a PC, an anemometer, and a digital storage oscilloscope (TDS 2012C, Tektronix Inc., Beaverton, OR, USA), which can measure, display and record an output voltage across an external load resistor in real time. An energy-collecting and -storing circuit was composed of a capacitor, a rectifier, resistors, a LED light source, a pedometer, and a sensor, which rectified the output voltage, stored electricity and powered the LED light source, the pedometer, and the sensor.

Table 1 lists the materials and the dimensions parameters of the harvester. The dimension parameters and the materials of the flexible spring and the spring rod were adopted based on the flutter characteristic in the theoretical model. The materials of the airfoil, the flexible spring, the square prism, the spring rod, and the piezoelectric patch were polylactic acid (PLA), spring steel, acrylic plate, spring steel, and Lead Zirconate Titanate (PZT-5H), respectively. A standard NACA 0012 was chosen as the airfoil profile of the harvester, which was fabricated by 3D printed PLA plastic and mounted by a spanwise aluminum shaft. The shaft was fitted with bearings at both ends, which could rotate around the shaft with the aim of emulating the plunge–pitch DOF of the airfoil in the airflow. A piezoelectric patch of PZT-5H was attached to the clamped end of the flexible spring to form the flexible-spring energy harvester. They were glued together using epoxy resin, and the bonding surface was assumed to be intact and free of gap. The harvester system could select various airfoils and dimensions parameters to adapt the placed working environment.

In this paper, the harvester systems were named according to the length and the thickness of the flexible spring in the following analyses. For example, EH-130-0.5 represented the energy harvester with a flexible spring, having a length of 130 mm and a thickness of 0.5 mm.

## 3. Results and Discussion

### 3.1. Aerodynamic Response Analyses

**Figure 3:**
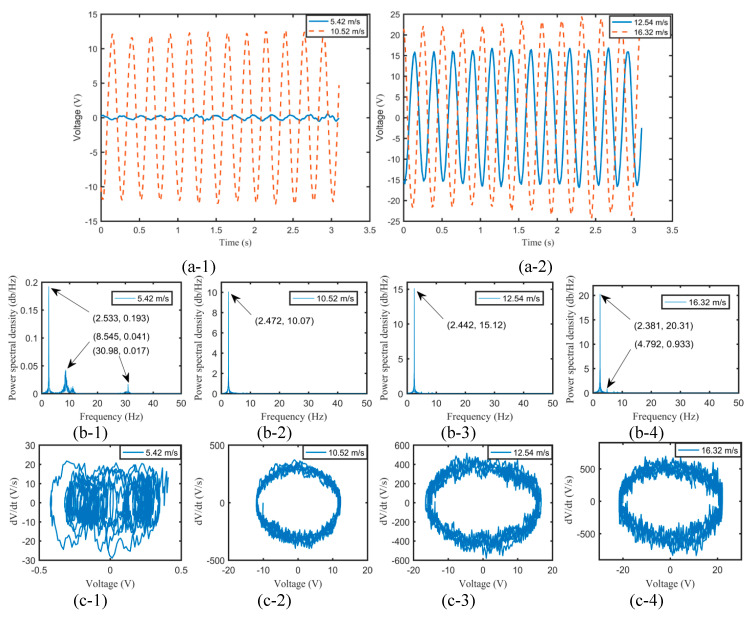
The time histories (**a**), the power spectral densities (**b**), and the phase portraits (**c**) of output voltages for EH-180-1 at 5.42, 10.52, 12.54, and 16.32 m/s.

The phenomenon of the limit-cycle oscillation of a harvester can occur at a certain airflow velocity that exceeded the critical flutter velocity. It turns into a flutter with a constant amplitude and self-excitation. According to the constitutive equation between the strain and the output voltage of a harvester, the voltage is proportional to the strain and the stress. Therefore, it can reflect and analyze quantitatively the aeroelastic response of the harvester. The open-circuit output voltage was used in this paper for evaluating the vibration response. It is noted that the cruising velocity of micro-airfoil aircrafts is usually between 10 and 35 m/s. However, the airflow velocity of the fabricated wind tunnel was adjusted from 0 to 22.73 m/s. Moreover, to protect and avoid the permanent damage of the harvesters, it should be also pointed out that the maximum airflow velocities of 14.48 and 16.32 m/s were adopted for the flexible springs with thicknesses of 0.5 and 1 mm. Figure 3 illustrates the time histories, the power spectral densities, and the phase portraits of output voltages for EH-180-1 at 5.42, 10.52, 12.54, and 16.32 m/s with the *Y*-axis representing the open-circuit output voltage of the harvester.

**Figure 4:**
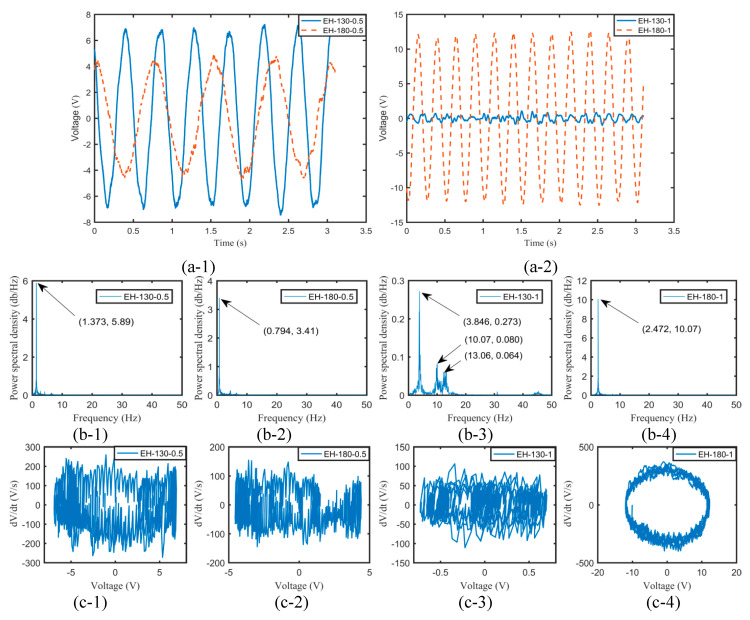
The time histories (**a**), the power spectral densities (**b**), and the phase portraits (**c**) of output voltages for various harvesters at 10.52 m/s.

As can be seen from Figure 3(a-1), the flexible-spring harvester EH-180-1 produced unstable output voltages, and chaos occurred concomitantly at 5.42 m/s. The power spectral density observed from Figure 3(b-1) also showed that the vibration response took place at three dominant frequencies of 2.533, 8.545, and 30.98 Hz, which demonstrated multi-frequency and multi-amplitude vibration. The chaos was also further confirmed by the phase portrait shown in Figure 3(c-1). The limit-cycle oscillation was not very smooth. The reason may be that the airfoil, the holder, and the fixture were fabricated by 3D printed PLA plastic. The thicknesses of the holder and the fixture were only 4 mm, and thus, the overall strength of the harvester was weaker compared with that of a conventional flutter harvester [42]. In addition, there existed a gap between the spring rod and the aluminum shaft during the installation process. The smaller aerodynamic force caused disturbance, and even buffeting occurred unexpectedly at a lower airflow velocity. However, increasing the airflow velocity augmented the aerodynamic force to act on the harvester. The structural damping of the harvester eventually turned from positive to negative, and thus a flutter took place at 10.52, 12.54, and 16.32 m/s. The obvious phenomenon of the flutter occurred at a higher airflow velocity and suppressed the unexpected disturbance to some extent, which also increased the stable output voltage and enhanced simultaneously the voltage amplitude. As shown in Figure 3(a-1,a-2), it was indicated that the amplitude of the quasi-periodic output voltage increased with the airflow velocity. The foundational vibration frequencies were almost the same at 10.52, 12.54, and 16.32 m/s, and the peak also increased gradually. The limit-cycle oscillations, as observed from Figure 3(c-2–4), appeared at a higher airflow velocity, and the vibration response with the voltage amplitude and the voltage velocity turned into violent with the airflow velocity. However, the vibration response of the harvester at a higher airflow velocity cannot be a completely periodic and simple limit-cycle oscillation, because the occurrence of disturbance and accidentally buffeting impaired the flutter and the limit-cycle oscillation.

The overall strength and stiffness of the harvester had a significant effect on the vibration response, and thus, the influence of the flexible springs on the aeroelastic vibration needed to be explored. Figure 4 illustrates the time histories, the power spectral densities, and the phase portraits of output voltages for various harvesters at 10.52 m/s.

It can be found from Figure 4(a-1) that although the harvesters EH-130-0.5 and EH-180-0.5 generated quasi-periodic output voltages, the buffeting phenomenon also occurred, and thus, the vibration at the peak amplitude was very complex and irregular. However, the harvester EH-180-1 could resist buffeting and thus produce stable and quasi-periodic output voltages. When the airflow velocity exceeded the critical flutter velocity, the longer and the thicker the flexible spring was, the better stability and the larger amplitude vibration of the harvester can be achieved. That may be because it owned a larger stiffness and thus could possess sufficient ability to sustain the same aerodynamic force. It can be recognized that the output voltage of EH-130-0.5 could be higher than that of EH-180-0.5 while the effect was opposite for the harvesters with a flexible spring having a thickness of 1 mm. It should be noted that a higher airflow velocity was required to overcome structural damping and attain to a flutter for EH-130-1 compared with that for EH-180-1. Therefore, multi-frequency chaotic vibration was observed from Figure 4(b-3) for EH-130-1. In Figure 4(c-1–4), the phase portrait indicated that limit-cycle oscillation occurred for EH-180-1 and the chaos phenomenon also took place for EH-130-0.5, EH-180-0.5, and EH-130-1 at 10.52 m/s.

**Figure 5:**
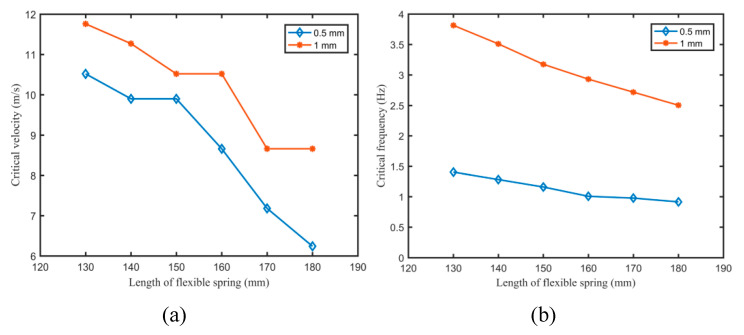
Variations of the critical velocity (**a**) and the critical frequency (**b**) of the harvester with the length of the flexible spring at thicknesses of 0.5 and 1 mm.

To further explore the influence of the flexible spring on the aerodynamic response, Figure 5 illustrates the variations of the critical velocity and frequency of the harvesters with the length of the flexible spring having thicknesses of 0.5 and 1 mm.

According to the equation $U_{c}∼\sqrt{Yh^{3}/\left(\rho l_{}^{3}\right)}$, where *U_c_* refers to the critical velocity; *Y*, ρ, and *l* are the elastic modulus, the density, and the length of the flexible spring, respectively. The critical flutter velocity was closely related to the length of the flexible spring. As can be observed from Figure 5a, the critical velocity of the flutter gradually decreased with the length of the flexible spring, but fluctuation occurred unexpectedly for EH-160-1. It should be noted that installation accuracies for different harvesters cannot always be completely guaranteed. For EH-130-1, the critical velocity of 11.76 m/s was greater than that (8.66 m/s) for EH-180-1. The critical velocities of the harvesters with a thickness of 1 mm were obviously higher than those of the harvesters with a thickness of 0.5 mm. The thicker the flexible spring was, the larger the elastic stiffness was, and the higher critical velocity of the harvester was required. The flutter frequency can be approximately calculated using the equation $\omega ∼\sqrt{\rho_{\infty }U^{2}/\left(\rho hl\right)}$, where ω refers to the critical frequency; $\rho_{\infty }$ is the airflow density; *h* represents the thickness of the flexible spring. The dependency of the critical frequency on the length of the flexible spring gradually decreased, and the downtrend for the harvesters with a thickness of 1 mm was higher than that for the harvesters with a thickness of 0.5 mm. For EH-130-0.5, the critical frequency of the harvester was 1.404 Hz, while it was 3.815 Hz for EH-130-1. Therefore, it can provide a basic theory for designing optimal parameters and attaining to the flutter condition of the harvester.

**Figure 6:**
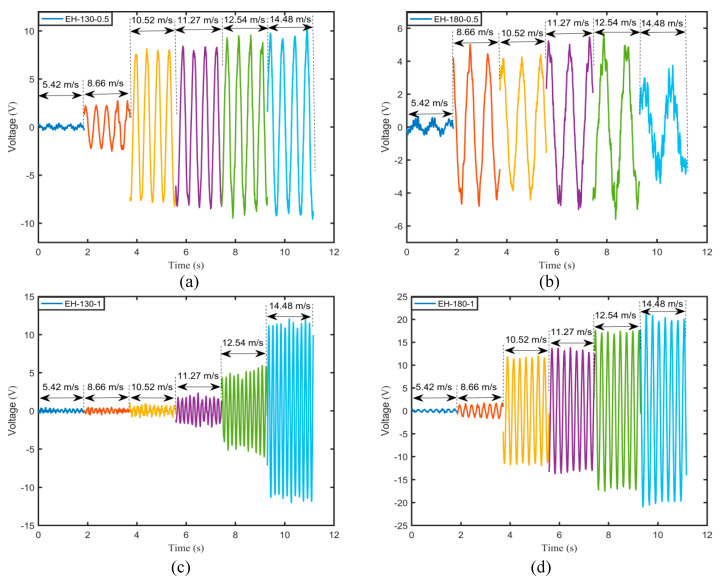
Variations of the time history of output voltage with airflow velocity for various harvesters: (**a**) EH-130-0.5; (**b**) EH-180-0.5; (**c**) EH-130-1; (**d**) EH-180-1.

The flutter velocities of the harvesters with various flexible springs were obtained by the above analyses. To express directly the aerodynamic responses of the harvesters, Figure 6 illustrates the variations of the time history of output voltage with airflow velocity for various harvesters.

It can be observed from Figure 6a that when the airflow velocity of 10.52 m/s exceeded the flutter onset velocity, i.e., 9.9 m/s for EH-130-0.5, the output voltage of the harvester rapidly increased and then rose slightly with the airflow velocity. For EH-180-0.5, the flutter velocity was lower than that for EH-130-0.5. However, as the airflow velocity was increased, the vibration response turned unstable and very complex, especially at the peak. It may be ascribed to the occurrence of buffeting, and thus the output voltage decreased rapidly at 14.48 m/s. The flutter onset velocities for EH-130-1 and EH-180-1 were higher than those for EH-130-0.5 and EH-180-0.5. However, when attaining to a flutter, the aerodynamic responses of the harvesters EH-130-1 and EH-180-1 were quite larger than those of EH-130-0.5 and EH-180-0.5. However, the critical frequency of EH-180-0.5 was lower than those of the other harvesters as observed from the relationship between the time history of output voltage and the airflow velocity.

Through the analyses of aerodynamic vibration response, a higher airflow velocity could lead to a flutter. However, the unsteady airflow velocity and the low stiffness of a harvester may cause the phenomenon of disturbance and even buffeting. To guarantee stable aerodynamic vibration and obtain a periodic voltage, it is wise to adopt longer and thicker flexible springs to form a harvester. In addition, a flutter and a limit-cycle oscillation could suppress unexpected disturbance to some extent. Furthermore, it enhanced the periodic vibration and improved the harvesting performance. Therefore, it is necessary to further explore the output voltage and power of the harvesters at different airflow velocities and the structural parameters of the flexible spring for the future field application.

### 3.2. Harvesting Performance Analyses

**Figure 7:**
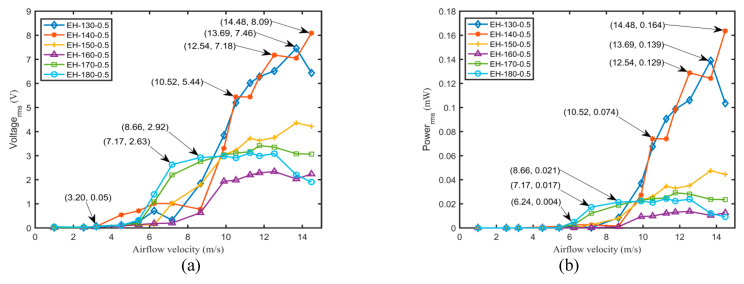
Variations of output voltage (**a**) and output power (**b**) with airflow velocity for various harvesters with a thickness of 0.5 mm and a resistance of 400 kΩ.

**Figure 8:**
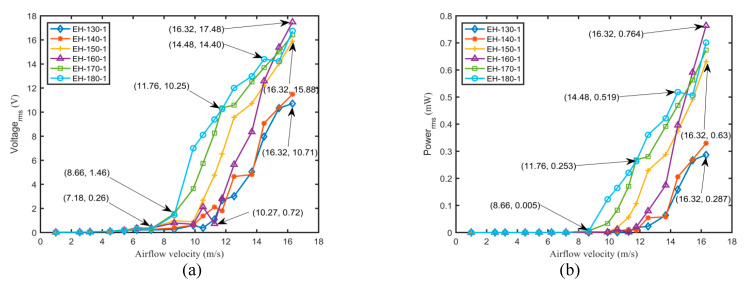
Variations of output voltage (**a**) and output power (**b**) with airflow velocity for various harvesters with a thickness of 1 mm and a resistance of 400 kΩ.

To explore the effects of the structural parameters of the flexible spring and airflow velocities on the output voltages and powers of different harvesters, the harvesting performances of the harvesters with a flexible spring under various conditions were thoroughly investigated by the experimental method. An external load resistor with a resistance of 400 kΩ that was connected to the piezoelectric patch was utilized for evaluating the harvesting performance in the following analyses. Figure 7 and Figure 8 illustrate the variations of output root-mean-square voltage and power with airflow velocity for various harvesters.

Figure 7a indicates that the output voltage first approached zero, increased rapidly, became flat gradually and eventually decreases with the airflow velocity for various harvesters. The flutter appeared with the increase of the airflow velocity. However, the output voltage of the harvester became flat gradually and even decreased slightly when exceeding the critical velocity. The cause may be attributed to the lower elastic stiffness of the harvesters EH-150-0.5, EH-160-0.5, EH-170-0.5, and EH-180-0.5, which meant that they could not adequately resist the larger aerodynamic force at a higher airflow velocity. The unstable vibration and the lower strain energy could not guarantee the increase of output voltage. However, for EH-130-0.5 and EH-140-0.5, the better harvesting performance can be achieved. For a harvester with a thickness of 0.5 mm, the output voltage of a shorter harvester obviously outperformed than that of a longer one. A maximum output voltage of 8.09 V and a peak output power of 0.164 mW was obtained for EH-140-0.5 at 14.48 m/s. Figure 7b demonstrates that the variations of output voltage and power were the same. Because of the higher internal resistance of the harvesters, high output voltages and low output powers were generated. The discrepancy of output power between EH-130-0.5 and EH-140-0.5 was quite small.

As can be observed from Figure 8a, the longer the flexible spring was, the higher output voltage was obtained at the same airflow velocity. A longer flexible spring owned a lower critical velocity and thus produced easily a larger vibration response under the same conditions. It also possessed a lower natural frequency that was easy to excite by an incoming airflow. It should be noted that the output voltage rapidly increased with airflow velocity under various harvesters. However, the decreasing tendency would not occur eventually as observed from various harvesters with a thickness of 0.5 mm. A thicker flexible spring could sustain the larger aerodynamic force generated by the airfoil and maintain a limit-cycle oscillation. Similarly, a maximum airflow velocity of 16.32 m/s was utilized for the harvesters with a flexible spring having a thickness of 1 mm. A maximum output root-mean-square voltage of 17.48 V and a peak power of 0.764 mW were obtained for EH-160-1 at 16.32 m/s. Figure 8b indicates that the output powers of the harvesters were near zero below the critical velocity. When exceeding the corresponding critical onset velocity, the output powers of the harvesters increased rapidly with the airflow velocity. The various structural parameters of the flexible spring exerted a significant influence on the output power of the harvester.

Compared with the output performance of the harvesters with a flexible spring having thicknesses of 0.5 mm, the overall performances of the harvesters with a flexible spring having a thickness of 1 mm were better at a higher airflow velocity. However, the overall performances of the harvesters with a flexible spring having a thickness of 1 mm were worse than those of the harvesters with a flexible spring having thicknesses of 0.5 mm at a lower airflow velocity. That is because the critical velocities of the harvesters with a flexible spring having a thickness of 1 mm were higher than those of the harvesters with a flexible spring having a thickness of 0.5 mm. Therefore, to harvest the better performance and promote the future field application of the harvester, it is sensible to choose a specific harvester according to the airflow velocity at the actual placed condition.

A comparison of output performances between the proposed harvester and previously reported harvesters was conducted, as shown in Table 2.

The previously reported harvesters adopted piezoelectric and electromagnetic-piezoelectric mechanisms, but they are all subjected to an incoming airflow. It should be pointed out that the power densities are defined as *P_ave_*/*A_m_* and *P_ave_*/*V_m_*, where *P_ave_* represents the average output power of the harvester; *A_m_* denotes the maximum side area of the entire harvester; *V_m_* is the maximum volume of the harvester. As can be known, the power densities of the proposed harvester were 14.71 and 2.94 μW/cm^3^, which demonstrated the superior harvesting performance over the others.

### 3.3. Field Application Testing

**Figure 9:**
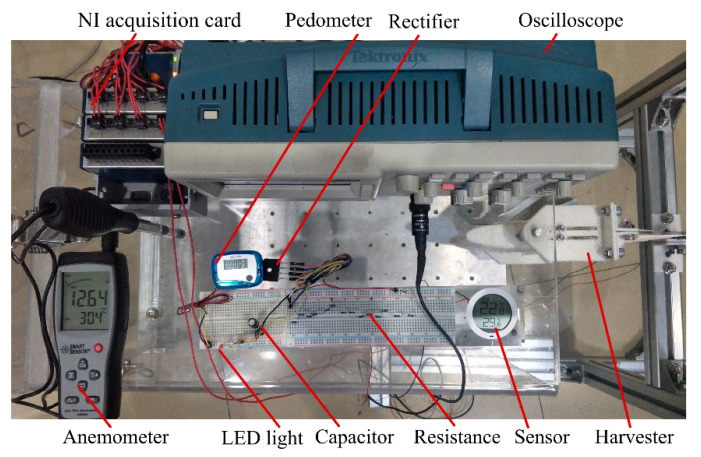
Photo of the energy-collecting and -storing circuit and the testing devices.

According to the above aerodynamic response and output performance analyses, the effects of the structural parameters of a flexible spring, airflow velocities, and external resistances on the aerodynamic responses and the output voltages and powers of various harvesters were obtained. The field application testing was required for validating the actual harvesting performance and considering the future practical application in the ambient environment. A standard energy-collecting and -storing electric circuit was utilized in this paper for evaluating the harvesting performance. A LED light source (working voltage: 2 V; power: 40 mW), a pedometer (initially powered by a 1.5 V cell button battery; capacity: 80 mAh), and a temperature and humidity sensor (powered by a 1.5 V AAA battery; capacity: 1300 mAh) (Xiaomi Communication Technology Co. Ltd., Beijing, China) were adopted as a mean to test and verify the output performance of the harvester. Figure 9 shows the energy-collecting and -storing circuit and the test devices.

**Figure 10:**
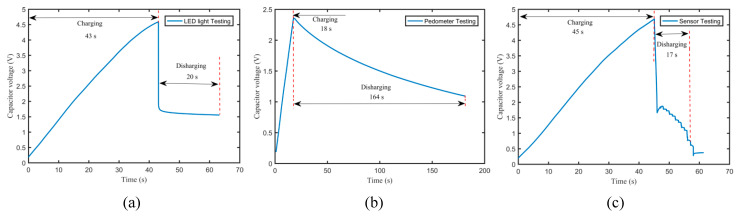
Variation of the capacitor voltage with time for EH-180-1 at 16.32 m/s: (**a**) capacitor voltage for LED light testing; (**b**) capacitor voltage for pedometer testing; (**c**) capacitor voltage for sensor testing.

As can be seen from Figure 9, the output voltage was generated by the harvester and input to the rectifier. Then, the rectified voltage charged directly the capacitor (nominal voltage 6.3 V, capacitance 1000 μF). After charging, the verification experiments of powering the LED light source, the pedometer, and the sensor via the capacitor were performed. Figure 10 illustrates the variation of the capacitor voltage with time for EH-180-1 at 16.32 m/s. As can be known from Figure 10a, when the rectifier was connected to the capacitor for attaining to 4.59 V, the charging time was 43 s, and 53.1% of the rated capacity was achieved. When the charging capacitor directly powered the LED light source, the working time could be 20 s. For the pedometer testing, the charging capacitor attaining to 2.36 V must be stopped for protecting the pedometer. When it powered a pedometer, the working operation could continue for 164 s, as observed from Figure 10c. For the sensor testing, the charging capacitor needed to be charged for 45 s to attain to a voltage of 4.69 V. When it powered the sensor that displayed the temperature and humidity in real time, the working time could be 17 s.

Through designing and constructing the standard energy-collecting and -storing circuit, the electricity generated by the harvester could charge the capacitor and then power the LED light source, the pedometer, and the sensor. If the harvester can work on and off in natural environment, it can drive continuously low-power electric equipment. However, the charging efficiency and the electric convection efficiency are relatively low. Therefore, it is urgent to exploit a higher-efficiency circuit, including a DC–DC converter with a regulator, for storing electricity and promoting future field applications. In addition, it is still necessary to carry out a reliability test in practical applications for guaranteeing reliable and stable operation.

## 4. Conclusions

This paper explores the aerodynamic responses and the output performancs of airfoil-based aeroelastic harvesters. A wind tunnel was developed, and several prototypes of harvesters with various flexible springs were fabricated. The effects of the structural parameters of the flexible spring, airflow velocities, and external resistances on the nonlinear aeroelastic response and the harvesting performance of the harvesters were experimentally investigated. The experimental results showed that the flutters of the harvesters were generated and limit-cycle oscillation was observed at a higher airflow velocity. For the flexible spring thickness of 1 mm, the aerodynamic responses and the output performances of the harvesters with a longer flexible spring were better than those of the harvesters with a shorter flexible spring. However, for the flexible spring thickness of 0.5 mm, the aerodynamic responses and the output performances of the harvesters with a longer flexible spring were worse than those of the harvesters with a shorter flexible spring. The overall performances of the harvesters with a shorter flexible spring having a thickness of 1 mm outperformed those of the harvesters with a shorter flexible spring having a thickness of 0.5 mm at a higher airflow velocity, while the tendency was opposite at a lower airflow velocity. A maximum output root-mean-square voltage of 17.48 V and a power of 0.764 mW were harvested for EH-160-1 at 16.32 m/s, which was superior to the existing airfoil-based piezoelectric energy harvesters. Through the field application testing, the harvesting electricity can power an LED light source, a pedometer, and a sensor. When driving a pedometer, the normal working operation could continue for 164 s. The present work could offer an important experimental foundation for further exploring the output performance of the harvesters in micro or unmanned airfoil aircrafts.

## Figures and Tables

**Table 1:** The materials and dimensions parameters of the harvester.

Properties	Flexible Spring	Piezoelectric Patch	Square Prism	Airfoil	Spring Rod
Materials	Spring steel	Lead Zirconate Titanate (PZT-5H)	Acrylic plate	Polylactic acid (PLA)	Spring steel
Density, *ρ* (kg/m^3^)	7850	7500	1190	1250	7850
Young’s modulus, *E* (GPa)	210	66	30	3.5	210
Poisson’s ratio, *v*	0.29	0.3	0.39	-	0.29
Length, *l* (mm)	130 to 180	40	100	-	100
Width, *b_w_* (mm)	30	20	4	-	1
Thickness, *h* (mm)	0.5 to 1	0.2	4	-	-
Permittivity component, *ε* (nF/m)	-	13	-	-	-
Piezoelectric constant, *d*_31_ (C/N)	-	−274	-	-	-
Mass of the harvester, *m_T_* (g)	-	-	-	540	-
Mass of the airfoil, *m* (g)	-	-	-	380	-
Span, *s* (mm)	-	-	-	100	-
Semichord, *b* (mm)	-	-	-	126	-

**Table 2:** Comparison of output performance between the previous and proposed harvesters.

Reference	Mechanism	Airflow Velocity (m/s)	Average Power (μW)	Power Density (μW/cm^2^)	Power Density (μW/cm^3^)	Configuration
Zhao et al. [41]	Piezoelectric	6	100	3.7	0.71	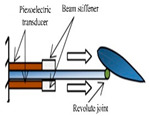
Abdelkefi et al. [42]	Piezoelectric	12.59	300	-	-	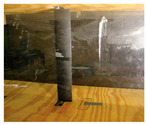
Cheng et al. [56]	Piezoelectric	42.4	13.06	4.66	-	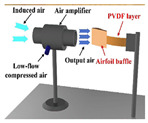
Iqbal et al. [57]	Electromagnetic-piezoelectric	6	11.2	0.16	0.04	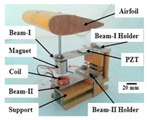
The proposed harvester	Piezoelectric	16.32	764	14.71	2.94